# Fabrication, Thermal Conductivity, and Mechanical Properties of Hexagonal-Boron-Nitride-Pattern-Embedded Aluminum Oxide Composites

**DOI:** 10.3390/nano12162815

**Published:** 2022-08-16

**Authors:** Hyesun Yun, Min-Gi Kwak, KeumHwan Park, Youngmin Kim

**Affiliations:** Display Research Center, Korea Electronics Technology Institute, Seongnam 13509, Korea

**Keywords:** aluminum oxide, hexagonal boron nitride, thermal conductivity, filler alignment

## Abstract

As electronics become more portable and compact, the demand for high-performance thermally conductive composites is increasing. Given that the thermal conductivity correlates with the content of thermally conductive fillers, it is important to fabricate composites with high filler loading. However, the increased viscosity of the composites upon the addition of these fillers impedes the fabrication of filler-reinforced composites through conventional methods. In this study, hexagonal-boron-nitride (h-BN)-pattern-embedded aluminum oxide (Al_2_O_3_) composites (Al/h-BN/Al composites) were fabricated by coating a solution of h-BN onto a silicone-based Al_2_O_3_ composite through a metal mask with square open areas. Because this method does not require the dispersion of h-BN into the Al_2_O_3_ composite, composites with high filler loading could be fabricated without the expected problems arising from increased viscosity. Based on the coatability and thixotropic rheological behaviors, a composite with 85 wt.% Al_2_O_3_ was chosen to fabricate Al/h-BN/Al composites. The content of the Al_2_O_3_ and the h-BN of the Al/h-BN/Al-1 composite was 74.1 wt.% and 12.8 wt.%, respectively. In addition to the increased filler content, the h-BN of the composite was aligned in a parallel direction by hot pressing. The in-plane (*k_x_*) and through-plane (*k_z_*) thermal conductivity of the composite was measured as 4.99 ± 0.15 Wm^−1^ K^−1^ and 1.68 ± 0.2 Wm^−1^ K^−1^, respectively. These results indicated that the method used in this study is practical not only for increasing the filler loading but also for achieving a high *k_x_* through the parallel alignment of h-BN fillers.

## 1. Introduction

Light-weight and electrically insulating polymers are widely used as packing materials for electronics [1]. However, the very low thermal conductivity (0.1–0.5 Wm^−^^1^ K^−^^1^) [2] of the polymers hampers their application as thermal interface materials (TIMs), which require both high insulation resistance and high thermal conductivity. To enhance the thermal conductivity of polymer composites, many researchers have endeavored to incorporate ceramic fillers into polymers [2,3,4]. Because of its low price, spherical shape, excellent insulating resistance [3], and good thermal conductivity (~30 Wm^−^^1^ K^−^^1^) [2], aluminum oxide (Al_2_O_3_) has been extensively used as a filler for the manufacturing of TIMs. To improve thermal conductivity further, hybrid filler systems have been prepared by incorporating fillers with higher thermal conductivity into Al_2_O_3_ composites, wherein increased filler packing density and the formation of heat pathways were expected [5]. Hexagonal boron nitride (h-BN) has an in-plane thermal conductivity that reaches 550 Wm^−1^ K^−1^ [6] due to the strong in-plane σ-bonds stemming from the sp^2^-hybridized boron and nitrogen atoms. Some studies [5,7,8,9,10,11] showed that the thermally conductive pathways formed by random bridging of Al_2_O_3_ and BN platelets increased the thermal conductivity of their composites. Despite this synergetic effect, the increased viscosity resulting from the addition of BN platelets deteriorates the processability, limiting the filler loading of the composites [7]. Given that with a greater filler content, the thermal conductivity of the composites is accordingly higher, it is crucial to attain higher filler loading while securing processability [12,13].

Owing to their excellent flexibility, high thermal stability, good wettability, and outstanding weatherability [14], polysiloxanes, also known as silicone rubbers (SRs), are widely used as polymer matrixes for filler-reinforced composites. Considering the low energy barrier for Si–O–Si rotation [15,16,17], SR composites are flexible even when highly loaded with filler. Ouyang et al. [18] achieved a flexible SR composite containing 55 vol% of Al_2_O_3_ without aggregation, owing to the strong hydrogen bond between Al_2_O_3_ and the silicone rubber. The thermal conductivity was 1.53 Wm^−1^ K^−1^. Yan et al. [19] used hybrid BNNS@Al_2_O_3_ fillers to fabricate a flexible SR composite with a filler content of 30 wt.%. The in-plane (*k_x_*) and through-plane (*k_z_*) thermal conductivity of the composite was 2.86 Wm^−1^ K^−1^ and 0.89 Wm^−1^ K^−1^, respectively, and these high values were attributed to the connection of BNNS by Al_2_O_3_. Although surface modification of fillers is one way to achieve composites with high filler loading, the lack of defects on BN surfaces has made it challenging to modify BN fillers [20,21,22,23]. In our previous study [24], highly filler-loaded composites were fabricated without having to disperse multilayer graphene into the composites. In addition, hot pressing further increased the thermal conductivity by aligning the graphene in a parallel direction [25]. In this study, h-BN-pattern-embedded Al_2_O_3_ composites (Al/h-BN/Al composites) were fabricated by coating an h-BN solution onto a silicone-based Al_2_O_3_ composite through a metal mask with square open areas. The content of h-BN in the Al/h-BN/Al composites was adjusted through the various open areas of the metal masks. Given that a methyl ethyl ketone solution with h-BN platelets was used to produce h-BN patterns, it was not necessary to disperse h-BN into the viscous Al_2_O_3_ composites. After investigating the thixotropic rheological behaviors [26] and coatability of three Al_2_O_3_ composites, a composite with 85 wt.% Al_2_O_3_ was chosen to fabricate Al/h-BN/Al composites. Finally, an intact Al/h-BN/Al-1 composite was obtained, and its thermal conductivity, volume resistivity, and mechanical properties were investigated.

## 2. Materials and Methods

### 2.1. Materials

Vinyl terminated polysiloxanes (VP-1000), hydride terminated polysiloxanes (HPP-3036), a platinum catalyst, and an inhibitor were purchased from HRS (Pyeongtaek, Korea). Monohydroxy terminated poly(dimethylsiloxane) (Mn ~ 4670) was purchased from Sigma-Aldrich Korea Ltd. (Yongin, Korea). Methyl ethyl ketone (MEK, 99.5%) was purchased from Samchun Chemicals (Pyeongtaek, Korea). The h-BN, with average lateral sizes of 11 μm and 31 μm, was purchased from Unitek Corp. (Incheon, Korea). The Al_2_O_3_, with mean diameters of 5 μm and 45 μm, was purchased from Denka Korea, Co., Ltd. (Seoul, Korea). All chemicals were used as received. The 300 μm-thick metal masks with square open areas of 3 mm × 3 mm, 5 mm × 5 mm, and 7 mm × 7 mm were purchased from Samborn Screen (Ansan, Korea). The space between patterns in the metal masks was 1 mm.

### 2.2. Instrumentation

An amplitude sweep test, a three interval thixotropy test (3ITT) and complex viscosity measurement were carried out using a rheometer (MCR 702, Anton Paar Korea Ltd, Seoul, Korea). A laser flash analysis was performed using a laser flash analyzer (LFA, Netzsch Korea Ltd, Paju, Korea). The specific heat capacity (Cp) of the samples was measured using a differential scanning calorimeter (DSC 214, Netzsch Korea Ltd, Paju, Korea). A tensile test was performed by pulling the sample along the *z*-axis at a pulling rate of 50 mm/min using a universal testing machine (HZ-1003A/B(1T), MMS Tech, Bucheon, Korea). The measurement of the volume resistivity was carried out using a high resistance meter (HP 4339B) equipped with a resistivity cell (HP 16008B). For measuring the volume resistivity, a current with a voltage of 500 V was applied to a sample for 60 s. The surface morphology of a torn sample was analyzed by scanning electron microscopy (SEM). This analysis was carried out using a scanning electron microscope (JSM 6390, JEOL Korea Ltd, Seoul, Korea) at an acceleration voltage of 20 KV.

### 2.3. Compositions of Thermally Conductive Composites

A silicone resin was produced by mixing vinyl terminated polysiloxane (1.87 g), hydride terminated polysiloxane (0.13 g), monohydroxy terminated poly(dimethylsiloxane) (0.2 g), a platinum catalyst (0.01 g), and an inhibitor (0.01 g). The h-BN mixture consisted of 11 μm h-BN (1.5 g) and 31 μm h-BN (3.5 g). The Al_2_O_3_ mixture was composed of 45 μm Al_2_O_3_ (15.4 g) and 5 μm Al_2_O_3_ (6.6 g). All compositions listed in Table 1 were admixed using a Thinky mixer (ARE-250, Thinky Corporation, Tokyo, Japan) for 5 min to produce the composites. The Al50 and Al/BN composites were bar-coated onto a release film and cured at 150 °C for 30 min to produce 1 mm-thick samples for the thermal diffusivity measurement.

### 2.4. An Amplitude Sweep Test and a 3ITT

To determine the limit of the linear viscoelastic (LVE) behavior of the samples, the storage modulus (G′) and loss modulus (G″) were measured by applying shear strain from 0.001% to 10% to each sample; samples were placed between two plates with a 1 mm gap between them at a constant frequency of 5 Hz. The thixotropic behavior of the composites was evaluated by a 3ITT. Shear strain of 0.001% was applied in the first interval (for 60 s) and third intervals (for 40 s), and shear strain of 1% was applied in the second interval (for 20 s). During the 3ITT experiment, the complex viscosity of the composites was measured.

### 2.5. Fabrication of Al/h-BN/Al Composites

**Figure 1:**
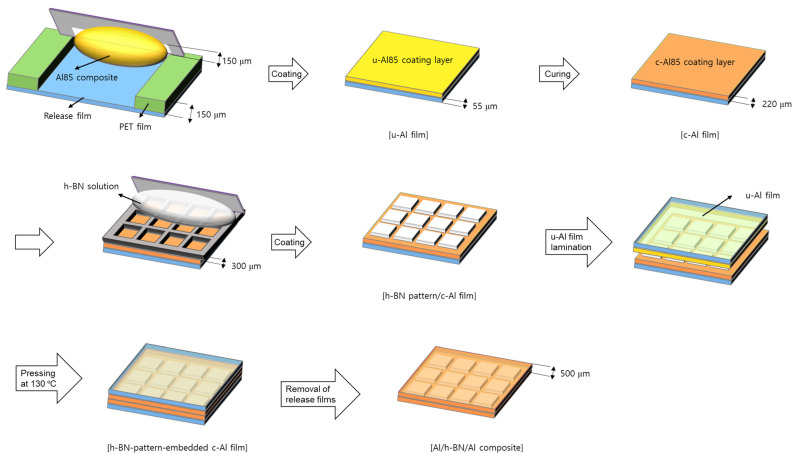
Schematic illustration of the fabrication process of Al/h-BN/Al composites.

The fabrication process of the Al/h-BN/Al composites is illustrated in Figure 1. First, the h-BN mixture (6.0 g) was admixed with MEK (6.0 g) to prepare an h-BN solution with a solid content of 50 wt.%. The Al85 composite outlined in Table 1 was coated onto a release film using a doctor blade with a 150 μm gap to yield a u-Al film, which was subsequently heated at 150 °C for 10 min to produce a 220 μm-thick Al85 layer on the release film (c-Al film). The as-prepared h-BN solution was then knife-coated onto the obtained c-Al film through a metal mask with square open areas and dried at 150 °C for 15 min to produce an h-BN pattern/c-Al film. The product was then laminated with the u-Al film, and was subsequently pressed under a pressure of 35 MPa at 130 °C for 30 min to yield an h-BN-pattern-embedded c-Al film. After the top and bottom release films were removed, a 500 μm-thick Al/h-BN/Al composite was obtained. The thickness of the h-BN patterns of the product was measured as 130 μm using a scanning electron microscope. The Al/h-BN/Al composites fabricated using metal masks with square open areas of 3 mm × 3 mm, 5 mm × 5 mm, and 7 mm × 7 mm are denoted as Al/h-BN/Al-1, Al/h-BN/Al-2, and Al/h-BN/Al-3, respectively.

### 2.6. Thermal Diffusivity Measurement

The as-prepared composites were punched into 25 mm circles and 10 mm × 10 mm squares for an in-plane and through-plane thermal diffusivity measurements. Both the top and bottom sides of the cut samples were coated with graphite via spray coating. After the samples were placed on a stage, their in-plane and through-plane thermal diffusivity was measured by irradiating them with a xenon lamp with a maximum energy pulse of 10 J/s. The pulse width was set as 300 μs and 180 μs for in-plane and through-plane thermal diffusivity measurements, respectively.

## 3. Results and Discussion

**Figure 2:**
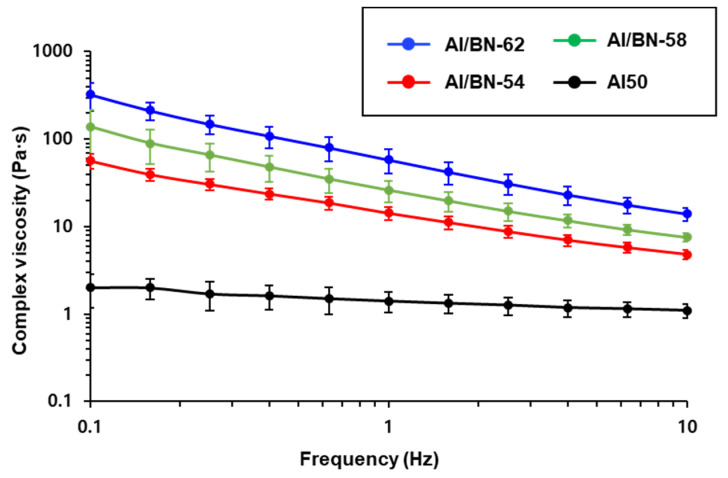
The frequency dependence of the complex viscosity of the Al50 and Al/BN composites.

Silicone-based Al50 and Al/BN composites containing various h-BN fillers were prepared according to the compositions outlined in Table 1. Given that the viscosity of the composites increases with increasing filler content, the effect of the amount of h-BN fillers on the complex viscosity was investigated through rheometry. The complex viscosity of the Al/BN composites increased with increasing h-BN loading, as shown in Figure 2. For instance, the complex viscosity measured at 0.1 Hz increased from ~2 Pa·s (for Al50) to ~57 Pa·s (for Al/BN-54) because of the higher h-BN content of the latter composite. The complex viscosity of the composites was sensitive to the amount of h-BN due to its planar structure. The complex viscosity of all Al/BN composites decreased with increasing frequency because the random orientation of fillers at low shear rates aligned in the direction of the flow at higher shear rates, leading to a decrease in flow resistance (shear thinning) [4].

The effect of h-BN content on the thermal conductivity of the composites was investigated. Because of the anisotropic thermal conductivity of h-BN [6], both *k_x_* and *k_z_* of the 1 mm-thick composites were calculated using the thermal diffusivity (α) obtained through a laser flash method based on Equation (1) [27].

*k* = α × ρ × Cp (1)

where ρ is density and Cp is specific heat.

**Figure 3:**
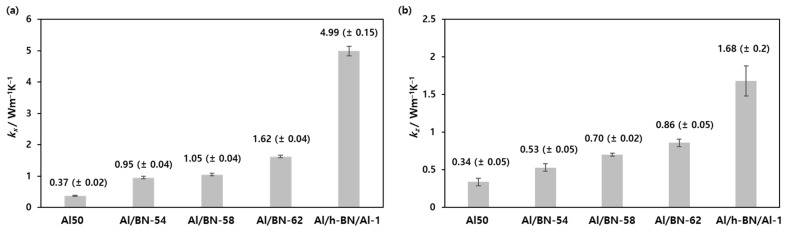
(**a**) *k_x_* and (**b**) *k_z_* of the Al50, Al/BN, and Al/h-BN/Al-1 composites.

The *k_x_* and *k_z_* values of the composites increased with increasing h-BN content, and *k_x_* was more sensitive to the amount of h-BN than *k_z_*, as shown in Figure 3. Given that the test specimens were prepared by bar-coating, the h-BN fillers partially aligned in a parallel direction and thus, *k_x_* was higher than *k_z_* [4,28,29,30,31]. The *k_x_* and *k_z_* values of the Al/BN-62 composite reached 1.62 ± 0.04 Wm^−1^ K^−1^ and 0.86 ± 0.05 Wm^−1^ K^−1^, respectively.

**Figure 4:**
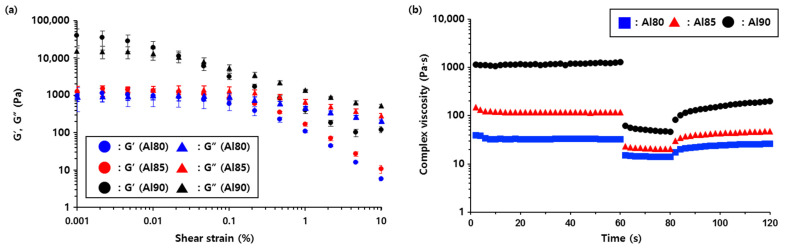
(**a**) Amplitude sweep and (**b**) 3ITT flow curves of the Al80, Al85, and Al90 composites.

Because the viscosity of the composites increased drastically with increasing h-BN content, it is challenging to produce Al/BN composites with >62 wt.% fillers through conventional methods. To address this issue, Al/h-BN/Al composites were fabricated through a sequential coating process in this study. Because a solution of h-BN was coated onto a silicone-based Al_2_O_3_ composite layer through a metal mask and laminated with another silicone-based Al_2_O_3_ composite by hot pressing (Figure 1), this fabrication method could be used to circumvent the use of highly viscous Al_2_O_3_/h-BN composites to attain high thermal conductivity [24]. To fabricate a Al_2_O_3_ composite layer that would be used as a substrate for h-BN patterns, three types of composites with 80 wt.% Al_2_O_3_ (Al80), 85 wt.% Al_2_O_3_ (Al85), and 90 wt.% Al_2_O_3_ (Al90) were prepared. Their thixotropic rheological behaviors were investigated through a 3ITT [32]. An amplitude sweep test was first performed to determine the limit of the LVE of the composites [33]. The G′ and G″ of each sample were measured under shear strain over a range from 0.001% to 10%, as shown in Figure 4a. The G′ value of the composites increased with increasing Al_2_O_3_ content, indicating that the three-dimensional networks of the fillers were extended. Given that the crossover point at which G′ = G″ increased from 0.005% (for Al80) to 0.02% (for Al90) with increasing Al_2_O_3_ fillers, the precipitation of Al_2_O_3_ fillers could be expected in the Al composites with fewer fillers over long-term storage. The limits of the LVE amplitude of the Al80, Al85, and Al90 composites were determined as 0.02%, 0.01%, and 0.005%, respectively. With the limits of the LVE in hand, the thixotropic rheological behaviors of the composites were investigated by a 3ITT (Figure 4b), where the first and the third intervals reflected “stabilization” and “recovery”, respectively, and the second interval mimicked the “coating process” [34,35]. The shear strain for the first and third intervals was set as 0.001%, and that for the second interval was set as 1% in the 3ITT experiments. The complex viscosity of the composites was stable in the first interval, which indicated that their structures remained undamaged. In the second interval, the high shear strain led to the deformation of the structures, and the complex viscosity drastically decreased. This shear-thinning behavior observed during the second interval was more noticeable with the Al90 composite because more fillers aligned in the direction of the flow. In the third interval, its complex viscosity increased with the partial recovery of the deformed structures, despite the degree of recovery becoming lower with increasing filler content.

**Figure 5:**
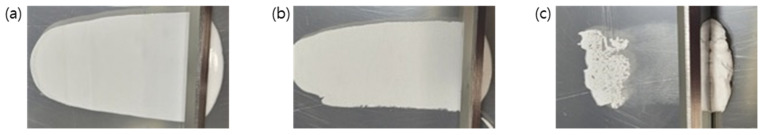
Digital photos of the coated (**a**) Al80, (**b**) Al85, and (**c**) Al90 composites on a release film.

**Figure 6:**
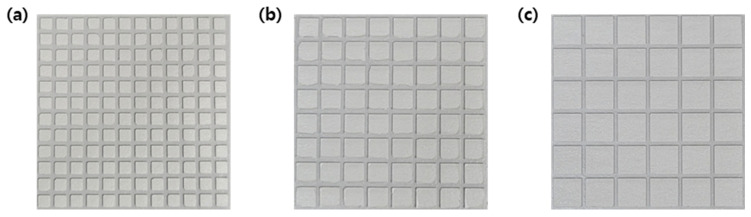
Digital photos of h-BN pattern/c-Al films fabricated using the metal masks with open areas of (**a**) 3 mm × 3 mm, (**b**) 5 mm × 5 mm, and (**c**) 7 mm × 7 mm. The space between patterns was 1 mm.

Next, the coatability of the Al80, Al85, and Al90 composites was evaluated by coating them onto a release film. While the Al80 and Al85 composites produced a coating layer, the Al90 composite failed to wet the release film because its viscosity was presumably too high (Figure 5). As disclosed by 3ITT experiments, the complex viscosity of the Al90 composite was higher than that of the other samples under shear strain ranging from 0.001% to 1%. Given that the thermal conductivity is correlated with the Al_2_O_3_ content, the Al85 composite, rather than the Al80 composite, was chosen to fabricate a substrate for the h-BN patterns. The u-Al85 composite layer of the u-Al film was cured at 150 °C to produce a 220 μm-thick c-Al85 composite layer of a c-Al film (Figure 1). The u-Al85 and c-Al85 samples are denoted as uncured Al85 and cured Al85, respectively, in this study. An h-BN solution was then knife-coated onto the c-Al film through a metal mask with square open areas and dried to yield an h-BN pattern/c-Al film. Three types of metal masks with various open areas were used to adjust the h-BN content of the final products (Figure 6). The larger the square open areas of the masks, the greater the h-BN content of the composites. The h-BN pattern/c-Al film was then laminated with the u-Al film and pressed at 130 °C under a pressure of 35 MPa. During this process, the u-Al85 composite filled the gaps between h-BN patterns, made contact with the bottom of the c-Al85 composite, and was cured to produce an h-BN-pattern-embedded Al film. After removing the top and bottom release films from the product, a 500 μm-thick Al/h-BN/Al composite was obtained. The thickness of the h-BN patterns was measured as 130 μm using a scanning electron microscope. The Al/h-BN/Al composites fabricated using metal masks with open areas of 3 mm × 3 mm, 5 mm × 5 mm, and 7 mm × 7 mm were denoted as Al/h-BN/Al-1, Al/h-BN/Al-2, and Al/h-BN/Al-3, respectively. The Al_2_O_3_ and h-BN content of the Al/h-BN/Al composites are summarized in Table 2. The h-BN content of the composites increased with increasing open areas of the metal masks. Given that it was challenging to produce Al/BN composites with >62 wt.% fillers because of the increased viscosity, the fabrication of Al/h-BN/Al composites with ≥12.8 wt.% h-BN and ≥70.2 wt.% Al_2_O_3_ is impressive.

**Figure 7:**
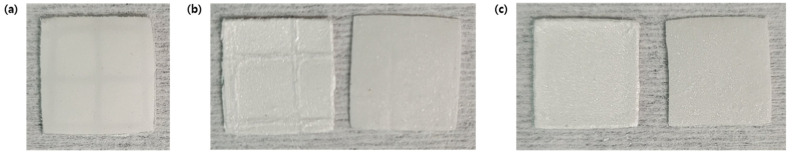
Digital photos of (**a**) Al/h-BN/Al-1, (**b**) Al/h-BN/Al-2, and (**c**) Al/h-BN/Al-3 composites after punching. Complete delamination was observed for the punched Al/h-BN/Al-2 and Al/h-BN/Al-3 composites.

Next, each Al/h-BN/Al composite was punched into a 10 mm × 10 mm square for the thermal conductivity measurement. While the Al/h-BN/Al-1 composite was isolated as an intact square (Figure 7a), complete delamination was observed for the other Al/h-BN/Al composites after punching (Figure 7b,c). Given that the top and bottom Al_2_O_3_ composites had created joints to hold the h-BN patterns tightly during hot pressing, the decreased joint areas with enlarged h-BN pattern areas caused the separation of h-BN interlayers in the Al/h-BN/Al-2 and Al/h-BN/Al-3 composites. Therefore, only the performance of the Al/h-BN/Al-1 composite was measured in this study. The *k_x_* and *k_z_* values of the Al/h-BN/Al-1 composite were determined as 4.99 ± 0.15 Wm^−1^ K^−1^ and 1.68 ± 0.2 Wm^−1^ K^−1^, respectively (Figure 3). The high *k_x_* value of the Al/h-BN/Al-1 composite was attributed to the h-BN alignment in a parallel direction during hot pressing [25]. This verified that the suggested technique not only increased the filler content but also aligned the h-BN in a parallel direction to attain high thermal conductivity of the composites. The volume resistivity of the Al/h-BN/Al-1 composite was 6.62 × 10^14^ Ω·cm, indicating that this product could be applicable as a TIM, which requires high thermal conductivity and excellent insulating properties.

**Figure 8:**
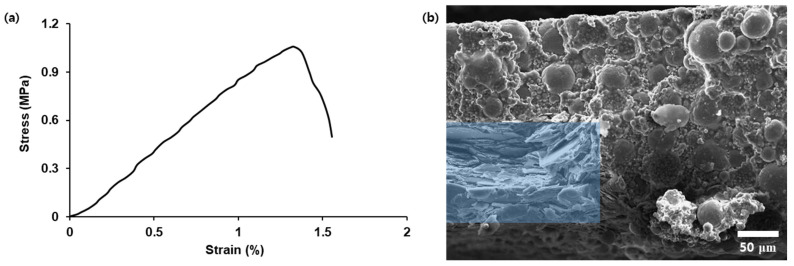
(**a**) Stress–strain curve of the Al/h-BN/Al-1 composite and (**b**) SEM image of the torn surface of the Al/h-BN/Al-1 composite after a tensile test. The blue area represents the stack of the aligned h-BN fillers in the composite.

The mechanical properties of the Al/h-BN/Al-1 composite were measured by pulling a 20 mm × 50 mm rectangle in the tensile direction until it was torn. Figure 8a shows the stress–strain curve of the Al/h-BN/Al-1 composite. The tensile strength, Young’s modulus, and elongation at break of the Al/h-BN/Al-1 composite were 1.04 ± 0.09 MPa, 95.0 ± 7.4 MPa, and 1.52 ± 0.26%, respectively. The morphology of the ruptured surface of the Al/h-BN/Al-1 composite obtained after the tensile test was analyzed using SEM, as shown in Figure 8b. The presence of the stack of the aligned h-BN fillers at the torn surface indicates that the initial crack was generated at the interface of the h-BN pattern and Al_2_O_3_ composite and then propagated into the Al_2_O_3_ composite. In addition, the craters observed in the Al_2_O_3_ composite show that the applied energy was transferred to the Al_2_O_3_ fillers and then dissipated through the pullout of the fillers [36].

## 4. Conclusions

In this study, composites with high filler loading were fabricated by coating an h-BN solution onto a silicone-based composite with 85 wt.% Al_2_O_3_ through metal masks with square open areas. This method could avoid the problems that arise from increased viscosity because the planar h-BN platelets were not added into the compositions of the Al_2_O_3_ composites. In addition, hot pressing induced alignment of h-BN in a parallel direction to increase *k_x_* of the composite further. Although the Al/h-BN/Al composites containing >15.8 wt.% h-BN were completely delaminated because of the decrease in areas jointed by Al_2_O_3_ composites, the Al/h-BN/Al-1 composite containing 74.1 wt.% Al_2_O_3_ and 12.8 wt.% h-BN remained undamaged after punching. The *k_x_* and *k_z_* values of the Al/h-BN/Al-1 composite were determined as 4.99 ± 0.15 Wm^−1^ K^−1^ and 1.68 ± 0.2 Wm^−1^ K^−1^, respectively. Furthermore, the volume resistivity of the composite was measured to be 6.62 × 10^14^ Ω·cm. In conclusion, the method suggested in this study has remarkable potential to realize composites with high thermal conductivity through the combination of high filler loading and parallel alignment of h-BN fillers.

## Figures and Tables

**Table 1:** The compositions of the Al50, Al80, Al85, Al90, and Al/BN composites.

Composites	Silicone Resin (g)	Al_2_O_3_ Mixture (g)	h-BN Mixture (g)	Filler Content (wt.%)
Al50	2.22	2.22		50
Al/BN-54	2.22	2.22	0.38	54
Al/BN-58	2.22	2.22	0.84	58
Al/BN-62	2.22	2.22	1.39	62
Al80	2.22	8.88		80
Al85	2.22	12.47		85
Al90	2.22	19.80		90

**Table 2:** The Al_2_O_3_ and h-BN contents of the Al/h-BN/Al composites.

Composites	Al_2_O_3_ (wt.%)	h-BN (wt.%)
Al/h-BN/Al-1	74.1	12.8
Al/h-BN/Al-2	71.6	15.8
Al/h-BN/Al-3	70.2	17.4

## Data Availability

Data sharing not applicable.

## References

[1] Wan Y.-J., Li G., Yao Y.-M., Zeng X.-L., Zhu P.-L., Sun R. (2020). Recent advances in polymer-based electronic packaging materials. Compos. Commun..

[2] Chen H., Ginzburg V.V., Yang J., Yang Y., Liu W., Huang Y., Du L., Chen B. (2016). Thermal conductivity of polymer-based composites: Fundamentals and applications. Prog. Polym. Sci..

[3] Ouyang Y., Bai L., Tian H., Li X., Yuan F. (2022). Recent progress of thermal conductive ploymer composites: Al_2_O_3_ fillers, properties and applications. Compos. Part A Appl. Sci. Manuf..

[4] Lim G., Bok G., Kim Y.-S., Kim Y. (2022). Fabrication of h-BN Filled Epoxy-Based Thermally Conductive Adhesive Tapes Containing Cyclic Carbonate-Terminated Oligomers. Electron. Mater. Lett..

[5] Zou D., Huang X., Zhu Y., Chen J., Jiang P. (2019). Boron nitride nanosheets endow the traditional dielectric polymer composites with advanced thermal management capability. Compos. Sci. Technol..

[6] Yuan C., Li J., Lindsay L., Cherns D., Pomeroy J.W., Liu S., Edgar J.H., Kuball M. (2019). Modulating the thermal conductivity in hexagonal boron nitride via controlled boron isotope concentration. Commun. Phys..

[7] Yetgin H., Veziroglu S., Aktas O.C., Yalçinkaya T. (2020). Enhancing thermal conductivity of epoxy with a binary filler system of h-BN platelets and Al2O3 nanoparticles. Int. J. Adhes. Adhes..

[8] Liu D., Ma C., Chi H., Li S., Zhang P., Dai P. (2020). Enhancing thermal conductivity of polyimide composite film by electrostatic self-assembly and two-step synergism of Al_2_O_3_ microspheres and BN nanosheets. RSC Adv..

[9] Wang Z., Meng G., Wang L., Tian L., Chen S., Wu G., Kong B., Cheng Y. (2021). Simultaneously enhanced dielectric properties and through-plane thermal conductivity of epoxy composites with alumina and boron nitride nanosheets. Sci. Rep.

[10] Yuan Y., Wu W., Hu H., Liu D., Shen H., Wang Z. (2021). The combination of Al2O3 and BN for enhancing the thermal conductivity of PA12 composites prepared by selective laser sintering. RSC Adv..

[11] Bian W., Yao T., Chen M., Zhang C., Shao T., Yang Y. (2018). The synergistic effects of the micro-BN and nano-Al2O3 in micro-nano composites on enhancing the thermal conductivity for insulating epoxy resin. Compos. Sci. Technol..

[12] Lim G., Bok G., Park S.-D., Kim Y. (2022). Thermally conductive hexagonal boron nitride/spherical aluminum oxide hybrid composites fabricated with epoxyorganosiloxane. Ceram. Int..

[13] Yun H., Han C.J., Park J.B., Kim Y. (2022). Thermal conductivity and mechanical properties of thermally conductive composites based on multifunctional epoxyorganosiloxanes and hexagonal boron nitride. Ceram. Int..

[14] Zalewski K., Chyłek Z., Trzciński W.A. (2021). A Review of Polysiloxanes in Terms of Their Application in Explosives. Polymers.

[15] Smith J.S., Borodin O., Smith G.D. (2004). A Quantum Chemistry Based Force Field for Poly(dimethylsiloxane). J. Phys. Chem. B.

[16] Weinhold F., West R. (2011). The Nature of the Silicon–Oxygen Bond. Organometallics.

[17] Poon L., Hum J.R., Weiss R.G. (2021). Neat Linear Polysiloxane-Based Ionic Polymers: Insights into Structure-Based Property Modifications and Applications. Macromol.

[18] Ouyang Y., Li X., Ding F., Bai L., Yuan F. (2020). Simultaneously enhance thermal conductive property and mechanical properties of silicon rubber composites by introducing ultrafine Al_2_O_3_ nanospheres prepared via thermal plasma. Compos. Sci. Technol..

[19] Yan H., Dai X., Ruan K., Zhang S., Shi X., Guo Y., Cai H., Gu J. (2021). Flexible thermally conductive and electrically insulating silicone rubber composite films with BNNS@Al_2_O_3_ fillers. Adv. Compos. Hybrid Mater..

[20] Bouville F., Deville S. (2014). Dispersion of Boron Nitride Powders in Aqueous Suspensions with Cellulose. J. Am. Ceram. Soc..

[21] Bashir A., Maqbool M., Lv R., Usman A., Guo H., Aftab W., Niu H., Liu M., Bai S.-L. (2021). Surface modified boron nitride towards enhanced thermal and mechanical performance of thermoplastic polyurethane composite. Compos. B. Eng..

[22] Muratov D.S., Kuznetsov D.V., Il’inykh I.A., Burmistrov I.N., Mazov I.N. (2015). Thermal conductivity of polypropylene composites filled with silane-modified hexagonal BN. Compos. Sci. Technol..

[23] Ren J., Stagi L., Innocenzi P. (2021). Hydroxylated boron nitride materials: From structures to functional applications. J. Mater. Sci..

[24] Bi J.C., Yun H., Cho M., Kwak M.-G., Ju B.-K., Kim Y. (2022). Thermal conductivity and mechanical durability of graphene composite films containing polymer-filled connected multilayer graphene patterns. Ceram. Int..

[25] Yu C., Gong W., Zhang J., Lv W., Tian W., Fan X., Yao Y. (2018). Hot pressing-induced alignment of hexagonal boron nitride in SEBS elastomer for superior thermally conductive composites. RSC Adv..

[26] Nan B., Gołębiewski P., Buczyński R., Galindo-Rosales F.J., Ferreira J.M.F. (2020). Direct Ink Writing Glass: A Preliminary Step for Optical Application. Materials.

[27] Lee H.-J., Lim G., Yang E., Kim Y.-S., Kwak M.-G., Kim Y. (2021). Thermally Conductive Film Fabricated Using Perforated Graphite Sheet and UV-Curable Pressure-Sensitive Adhesive. Nanomaterials.

[28] Liu J., Guo Y., Weng C., Zhang H., Zhang Z. (2020). High thermal conductive epoxy based composites fabricated by multi-material direct ink writing. Compos. Part A Appl. Sci. Manuf..

[29] Li J., Leng J., Jiang Y., Zhang J. (2021). Experimental characterization of 3D printed PP/h-BN thermally conductive composites with highly oriented h-BN and the effects of filler size. Compos. Part A Appl. Sci. Manuf..

[30] He Y., Li H., Luo F., Jin Y., Huang B., Qian Q. (2021). Bio-based flexible phase change composite film with high thermal conductivity for thermal energy storage. Compos. Part A Appl. Sci. Manuf..

[31] Luo F., Yang S., Yan P., Li H., Huang B., Qian Q., Chen Q. (2022). Orientation behavior and thermal conductivity of liquid crystal polymer composites based on Three-Dimensional printing. Compos. Part A Appl. Sci. Manuf..

[32] del-Mazo-Barbara L., Ginebra M.-P. (2021). Rheological characterisation of ceramic inks for 3D direct ink writing: A review. J. Eur. Ceram. Soc..

[33] Raymond Y., Thorel E., Liversain M., Riveiro A., Pou J., Ginebra M.-P. (2021). 3D printing non-cylindrical strands: Morphological and structural implications. Addit. Manuf..

[34] Toker O.S., Karasu S., Yilmaz M.T., Karaman S. (2015). Three interval thixotropy test (3ITT) in food applications: A novel technique to determine structural regeneration of mayonnaise under different shear conditions. Food Res. Int..

[35] Luo S., Weinell C.E., Okkels F., Østergård A.L., Kiil S. (2021). On-line, non-Newtonian capillary rheometry for continuous and in-line coatings production. J. Coat. Technol. Res..

[36] Bok G., Lim G., Park K., Kim Y. (2021). Mechanical properties and fracture toughness of fumed silica epoxy composites containing glycidyl terminated polysiloxanes. Ceram. Int..

